# Advance Approval of Outpatient Chemotherapy via Phone Call Optimizes Healthcare Delivery without Compromising Patient Satisfaction with Care

**DOI:** 10.3390/cancers13061337

**Published:** 2021-03-16

**Authors:** Patricia Marino, Rajae Touzani, Lorène Seguin, Jean Francois Moulin, Myriam Palomares, Maria-Antonietta Cappiello, Magali Provansal, Martine Vittot, Slimane Dermeche, Simon Launay, Anthony Goncalves, Anne Deborah Bouhnik, Gwenaelle Gravis

**Affiliations:** 1 Institut Paoli-Calmettes, SESSTIM, INSERM, IRD, Aix Marseille Université, 13009 Marseille, France; rajae.touzani@inserm.fr; 2 Department of Medical Oncology, Institut Paoli-Calmettes, 13009 Marseille, France; SEGUINL@ipc.unicancer.fr (L.S.); MOULINJF@ipc.unicancer.fr (J.F.M.); PALOMARESM@ipc.unicancer.fr (M.P.); CAPPIELLOM@ipc.unicancer.fr (M.-A.C.); PROVANSALM@ipc.unicancer.fr (M.P.); VITTOTM@ipc.unicancer.fr (M.V.); DERMECHES@ipc.unicancer.fr (S.D.); LAUNAYS@ipc.unicancer.fr (S.L.); GONCALVESA@ipc.unicancer.fr (A.G.); GRAVISG@ipc.unicancer.fr (G.G.); 3 INSERM U1068, CNRS UMR7258, CRCM, Aix-Marseille Université, 13009 Marseille, France; 4 INSERM, IRD, SESSTIM, Aix Marseille Université, 13009 Marseille, France; anne-deborah.bouhnik@inserm.fr

**Keywords:** chemotherapy, advance approval, phone call, satisfaction, healthcare organization

## Abstract

**Simple Summary:**

Patient satisfaction is a key parameter of care quality. Among oncology patients undergoing chemotherapy (CT), the long waiting times associated with frequent and prolonged consultations have been shown to be a major source of dissatisfaction. The aim of this study was to determine whether advance approval of outpatient CT via phone call the day before CT can optimize healthcare delivery without compromising patient satisfaction with care. Our results showed that the satisfaction level with physicians regarding technical skills, interpersonal skills, and availability were not decreased in patients who did not receive a face-to-face consultation with an oncologist the day of CT. We also found that waiting times were reduced for patients who were treated according to the advance approval procedure. These findings suggest that advanced approval of outpatient CT via phone call is a feasible alternative that does not compromise patient satisfaction with care.

**Abstract:**

Patient satisfaction is linked to the amount of time spent with the physician. At the same time, long waiting times in hospitals are a major source of patient dissatisfaction. The aim of this study was to determine whether advance approval of outpatient chemotherapy (CT) via phone call can optimize healthcare delivery without compromising patient satisfaction with care. Between 2013 and 2016, 343 patients with breast/gynecological cancer scheduled to undergo CT on day 8 and/or day 15 of the CT cycle were enrolled in a before–after study conducted in a French comprehensive cancer center. In the control group, 168 patients received a face-to-face consultation with an oncologist on the day of CT for approval of the upcoming CT session. In the intervention group, 175 patients received a phone call from a healthcare provider the day before CT, where assessment of toxicity from the previous CT session was recorded and submitted to an oncologist for approval of the upcoming CT session. At the end of the 6th CT cycle, patient satisfaction was evaluated using EORTC IN-PATSAT32. A total of 233 questionnaires were analyzed (response rate: 77.7%). Satisfaction with care was similar between the two groups. No differences in perceived health status were observed, but self-reported time in hospital was lower in the intervention group than in the control group (*p* = 0.007). Advance approval of outpatient CT via phone call is feasible and particularly relevant in the current context of immunotherapy development.

## 1. Introduction

Improving quality of care is a major concern for healthcare providers. The interest in measuring patients’ specific experience of the care pathway is fairly new and has grown rapidly in recent years. It is now well-established that patient satisfaction is a key parameter of care quality [1,2,3,4] and that it leads to better health outcomes [5,6]. Patient satisfaction has been shown to be influenced by multiple factors, including the clinical and sociodemographic characteristics of the patients themselves and the characteristics of health facilities [7,8,9]. Other factors include quality of physician–patient communication and time spent with the physician, both of which are major components of patient-centered care [10,11,12]. At the same time, the long waiting times associated with frequent and prolonged consultations have been shown to be a major source of dissatisfaction, notably among oncology patients undergoing chemotherapy (CT) [13,14,15,16,17].

With the development of targeted treatments and immunotherapies, an increasing number of patients receive CT in oncological outpatient departments. The typical procedure for weekly CT sessions includes the following steps: (1) on the day of intervention, an oncologist meets with the patient face-to-face and conducts a CT assessment based on biological test results and patient-reported adverse events since the previous CT session, (2) the oncologist decides whether or not to approve the upcoming CT session, and (3) when the session is approved, the patient waits in the hospital for the CT infusion to be prepared, sent to the outpatient clinic, and administered to him/her.

In 2015, our oncological outpatient clinic set up a new procedure consisting of advanced CT approval via phone call. This procedure includes the following steps: (1) the day before day 8 and/or day 15 of the CT cycle, a healthcare provider calls the patient at home to collect information on his/her adverse events experienced since the previous CT session, (2) this toxicity assessment is submitted the same day to an oncologist, who decides whether or not to approve the upcoming CT session, and (3) when the session is approved, the CT is prescribed and ordered the same day (the days before CT administration), and the patient is not required to meet face-to-face with the oncologist the day of the CT infusion. When the session is not approved, the patient is referred to the oncologist the day of CT.

The aim of this study was to determine whether advance approval of outpatient CT via phone call the day before CT can optimize healthcare delivery without compromising patient satisfaction with care, as compared to approval via face-to-face consultation on the day of CT.

## 2. Materials and Methods

### 2.1. Study Design

Between 2013 and 2016, we conducted a prospective single-center study in a Regional Comprehensive Cancer Center outpatient unit in France (Institut Paoli-Calmettes, IPC in Marseille).

Eligible patients had breast or gynecological cancer in the adjuvant or metastatic settings, for which there was an indication of weekly CT sessions. Other inclusion criteria were: being in good general condition (WHO performance status < 2), having no severe comorbidities, being less than 75 years old, not being included in a therapeutic trial, being scheduled to undergo CT on day 8 and/or day 15 of the CT cycle in the oncological outpatient unit, being able to speak and understand French, and being able to answer to a phone call. Those who met these criteria were invited to participate in the study and to sign a consent form during their first CT session.

Our study used a before–after design. In the “before” period (i.e., before the implementation of the new approval procedure, which corresponds to the control group), all patients scheduled to undergo CT in the oncological outpatient unit received a face-to-face consultation with an oncologist on the day of CT. Based on biological test results and patient-reported adverse events since the previous CT session, the oncologist could either renew the initial CT prescription, change CT dosage, delay the upcoming CT session, or discontinue treatment. Patients received CT only when approved by the oncologist.

In the “after” period (i.e., after the implementation of the new approval procedure, which corresponds to the intervention group), all patients received a face-to-face consultation with an oncologist only on day 1 of the CT cycle and then a phone call (no clinical assessment) from a healthcare provider the day before day 8 and/or day 15 of the CT cycle. A medical questionnaire was administered by phone to assess toxicity from the previous CT session, and then graded using the Common Terminology Criteria for Adverse Events (CTCAE version 3.0). This toxicity assessment was submitted the same day to an oncologist, who decided whether or not to renew the initial CT prescription (with no change in therapy) based on the patient’s biological test results (Hematology status assessed 48 h before) and the information collected in the questionnaire. The advice on how to manage possible toxicities and adapt the treatment was given by the healthcare provider after discussion with the referring oncologist.

When the CT session was approved, the pharmacy prepared the CT infusion the same day in order to reduce the waiting time of the patient on the day of CT infusion. The next day (i.e., the day of CT), patients received CT without needing to meet with the oncologist. Pulse and blood pressure were monitored by nurses before chemotherapy infusion.

All patients were allowed to meet face-to-face with the oncologist on the day of CT if they wished, even if the CT session had been approved the day before. Whenever biological results showed poor hematological recovery, the CT session was delayed. Patients who reported clinical toxicity over the phone were referred to the oncologist.

At the end of the 6th CT cycle, study patients were sent a self-administered questionnaire requesting information on socio-demographic characteristics, patient satisfaction with CT, and health-related quality of life. Waiting time, defined as the time between arrival in and departure from the oncological outpatient unit, was also recorded.

### 2.2. Self-Administered Questionnaire

The level of participant satisfaction with care received during CT cycles was assessed using the validated multidimensional scale EORTC IN-PATSAT32 [18]. This scale is composed of three subscales measuring: satisfaction with physicians (11 questions), satisfaction with nurses (11 questions), and satisfaction with organization of care (10 questions). Items are rated on a 5-level Likert scale as “poor,” “fair,” “good,” “very good,” or “excellent.” A higher score reflects a higher level of satisfaction.

Since the purpose of the new procedure was to remove the need for face-to-face consultation with an oncologist, our primary outcome was the EORTC IN-PATSAT32 subscale measuring satisfaction with physicians. This subscale is composed of 4 sub-scores: technical skills (3 questions), interpersonal skills (3 questions), information provision (3 questions), and availability (2 questions). The overall score ranges from 0 to 100, with a higher score representing greater satisfaction.

The level of trauma experienced during CT sessions was assessed using the Impact of Event Scale (IES) [19]. The IES is composed of 15 items scored from 0 (not at all) to 5 (often). It is composed of two sub-scales, one measuring intrusive thoughts about the trauma (score: 0–35) and the other measuring avoidance of situations reminiscent of the traumatic event (score: 0–40). An overall IES score greater than or equal to 26 signals the presence of post-traumatic stress disorder.

Quality of life was assessed using the EuroQol 5 Dimensions (EQ-5D). The five dimensions measured with this scale are: mobility, self-care, usual activities, pain/discomfort, and anxiety/depression.

Self-reported time in the oncological outpatient unit was calculated based on arrival and departure times declared by the patient.

Lastly, socio-demographic data were also included in the questionnaire (age, number of children, marital status, level of education, professional activity, financial situation).

The study was approved by the institutional review board of the Paoli-Calmettes Institute (IRB #COS 13-004). All patients signed an informed consent form before joining the study.

### 2.3. Statistical Analysis

The study sample was described according to sociodemographic and medical variables. Patient characteristics in the control and intervention groups were compared using the Chi-2 test. The overall score of satisfaction with physicians and its four sub-scores were expressed as mean and standard deviation (SD). The Student’s *t*-test and analysis of variance (ANOVA) test were used to compare the score of satisfaction with physicians according to different qualitative variables. The correlation score was calculated for continuous variables.

Missing data on self-reported time in the outpatient unit were imputed with average time calculated according to patient group and type of CT protocol used. A sensitivity analysis was performed with missing data on self-reported time. All the other variables were processed without taking into account the missing data.

A linear regression model was used to estimate the contributions of the different variables to the overall score of satisfaction with physicians. Each variable was entered in the univariate analysis to evaluate its effect on the score of satisfaction with physicians. Variables that were significant at the 20% level were entered in the multivariate model generated according to a progressive stepwise approach. The multivariate model was adjusted for patient group (control vs. intervention) and for self-reported time in the oncological outpatient unit. The significance threshold was set at 5%.

All analyses were performed using the statistical software package Stata/SE version 12.0.

## 3. Results

### 3.1. Population Characteristics

A total of 343 women participated in the study. Of these, 168 were included in the control group and 175 in the intervention group. A total of 233 participants returned the completed questionnaire (129 from the control group vs. 104 from the intervention group), leading to an overall response rate of 77.7% (83.2% for the control group vs. 71.7% for the intervention group). When the main medical characteristics of respondents and non-respondents were compared, non-respondents were found to be more likely than respondents to have cervical or uterine cancer (20.4% vs. 10.2%; *p* = 0.107) and to receive polychemotherapy (75.5% vs. 42.5%; *p* < 0.001).

Patient characteristics are presented in Table 1. The age of patients ranged from 27 to 85 years, and most patients (63.5%) were aged between 48 and 68 years. The majority of patients lived with a partner (59.5%), had no professional activity (55.6%), and had a satisfactory financial situation (45.8%). As regards medical data, most participants were women with breast cancer (50.7%), received mono-chemotherapy (57.5%), and 71.4% were in the metastatic stage. No significant differences in patient characteristics were observed between the control group and the intervention group. Participants waited an average of 4 h 48 min between arrival in and departure from the oncological outpatient unit. The difference in waiting time between the control group and the intervention group was statistically significant (5 h 10 min, 95% confidence interval (CI) (4 h 44 min–5 h 22 min) vs. 4 h 29 min, 95%CI (4 h 07 min–4 h 52 min); *p* = 0.021). In the sensitivity analysis where missing data were not imputed, we found similar results with a significant difference in waiting time between the control group and the intervention group (5 h 10 min, 95%CI (4 h 50 min–5 h 29 min) vs. 4 h 30 min, 95%CI (4 h 04 min–4 h 55 min), *p* = 0.012).

### 3.2. Patients’ Satisfaction

**Figure 1:**
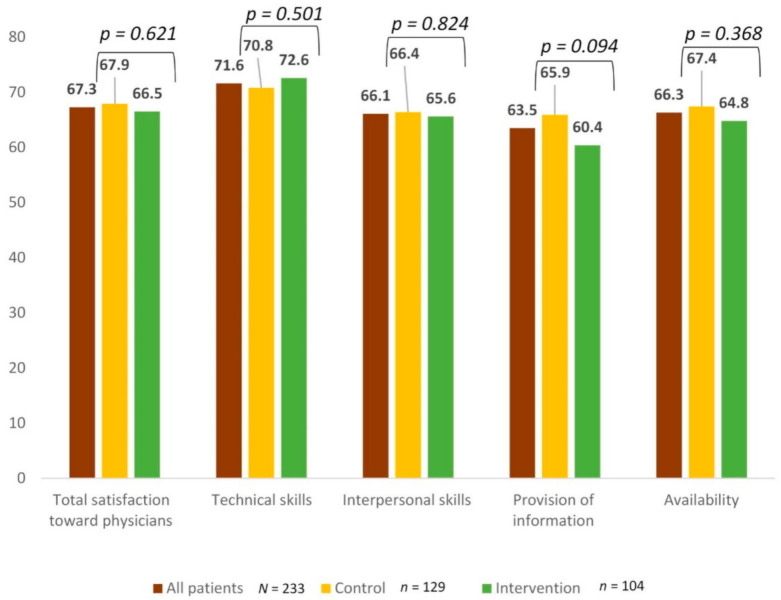
Average score of satisfaction with physicians for the whole sample and for each patient group (control vs. intervention).

Figure 1 shows the average overall score of satisfaction with physicians and its 4 associated sub-scores. The average satisfaction score was 67.3 (SD: 20.4) for the full sample, with no difference between the two groups of patients (67.9 vs. 66.5, *p* = 0.621). Satisfaction with technical skills, interpersonal skills, information provision, and availability of physicians was similar between the two groups.

The factors associated with satisfaction with physicians are presented in Table 2. The score of satisfaction with physicians was significantly higher in women who felt that the time spent in the oncological outpatient unit was shorter than expected (*p* = 0.007), women who felt that the time spent in the oncological outpatient clinic was adapted to the care received (*p* = 0.001), and women with a better quality of life (QOL) (*p* = 0.026). Satisfaction with physicians was not significantly associated with patient group or with self-reported time in the oncological outpatient unit.

The multivariate analysis is presented Table 3. After adjusting the model for patient group (control or intervention) and for self-reported time in the oncological outpatient unit, the score of satisfaction with physicians was 10.6 points (*p* = 0.044) higher in women aged 69 to 85 years than in women younger than 69 years. Similarly, the score of satisfaction with physicians was 8.9 points (*p* = 0.008) higher in women who felt that the time spent in the oncological outpatient unit was adapted to the care received.

## 4. Discussion

It is now established that patient satisfaction is an important predictor of care quality [1,2,3,4]. Patients tend to place great value on the time spent with physicians, which makes them feel that they receive the needed attention. At the same time, long waiting times, especially for scheduled appointments with physicians, are a major predictor of patient dissatisfaction [20,21]. The aim of this study was to determine whether advance approval of outpatient CT via phone call the day before CT can optimize healthcare delivery without compromising patient satisfaction with care, as compared to approval via face-to-face consultation on the day of CT. To date, few studies have described and assessed the implementation of a phone-based procedure for CT approval. Indeed, studies have most often demonstrated that phone calls constitute a safe and feasible strategy for the management of CT side effects [22,23,24,25], but few have examined their safety and feasibility for approval of outpatient CT [26,27,28], and only one analyzed the impact of advance CT approval on patient satisfaction [29].

Our study results indicate that patient satisfaction was high under the new procedure, as the overall score of satisfaction with physicians and each of its 4 sub-scores was above 60 (on a 0–100 possible range). The sub-scale measuring satisfaction with information provision had the lowest score in both patient groups.

The overall score of satisfaction with physicians was similar in the two patient groups. In particular, satisfaction with technical skills, interpersonal skills, and availability were not decreased in patients who did not receive a face-to-face consultation with an oncologist. This is an important finding given that the purpose of the new procedure was to remove the need for such consultation. Our results confirm the findings of the study by Dos Santos et al. [29], which highlighted high general score of satisfaction with care for patients included in a program aimed to anticipate the prescription of ambulatory CT.

As noted earlier, waiting time is an important aspect of patient satisfaction. In our study, it was significantly decreased in the intervention group compared to the control group (4.29 h vs. 5.10 h, *p* = 0.021). This decrease, however, was not as large as expected, which may be partly explained by the fact that making changes in the organization of care takes time. For instance, it may be that preparation of CT the day before intervention was inefficiently organized at the initial stage of implementation of the new procedure. At the time of the study, this new organization was not routinely implemented. This study, however, allowed us to modify the outpatient unit organization, and to date, the implementation of this new procedure resulted in waiting times that are significantly decreased: in 2016, more than 50% of patients had a waiting time of more than 2 h vs. 17.8% in 2020.

Interestingly, in the multivariate model, after adjusting for other variables, the only variable significantly associated with the score of satisfaction with physicians was the feeling that the time spent in the oncological outpatient unit was adapted to the care received. The satisfaction score was increased by 8.9 points in patients who felt that the time spent in the oncological outpatient clinic was adapted compared to those who did not (*p* = 0.008). By contrast, absolute time spent in the oncological outpatient unit did not impact satisfaction with physicians. We can conclude from this that patient satisfaction is impacted by perceptions of the appropriateness of waiting time relative to the care received, even when the waiting time was higher than expected. This finding contradicts the study by Bleustein et al. [21], in which longer waiting times reduced patient satisfaction with physicians.

Nevertheless, it should be emphasized that the new procedure for CT approval may not be adapted to all patients. In our study, 26.8% of patients reported feeling that face-to-face consultation with an oncologist was imperative before each CT session. Such patients should not be imposed a phone-based CT approval procedure that might increasetheir level of anxiety. However, Impact of Event Scale (IES) scores were similar between the two patient groups. Considering that this scale is correlated with anxiety and depression measures, it suggests that anxiety was not increased in the intervention group.

In addition to ensuring patient satisfaction, advanced approval of CT via phone call improves the effectiveness of outpatient and pharmaceutical services. This is especially important in a context where the availability of intravenous drugs and the number of patients receiving targeted treatments and immunotherapy are growing, making it increasingly difficult to prepare CT infusion in a timely manner.

From an economic perspective, such changes in the organization of care can improve the utilization of scarce resources. Thus, in the study by Coriat et al. [30], the implementation of a phone-based monitoring program for cancer patients receiving outpatient CT proved to be a cost-effective strategy (leading to a €201,468 decrease in hospital costs per year). In addition, by saving the time of patients, physicians, and pharmacists, phone-based monitoring improves both patient flow and the deployment of medical staff, thereby optimizing care delivery in the outpatient setting.

Lastly, from the perspective of the French healthcare insurance system, advance approval of outpatient CT via phone call can reduce unnecessary expenses by removing the need for medical transports when CT is not approved. In our study, no unscheduled hospital visits or additional visits to general practitioners were observed in the intervention group, indicating that the new procedure did not result in a transfer of costs from hospital to community care and, by implication, that adverse events were not underreported.

In our study, all phone calls were made by a junior physician. In the future, phone assessments for advance approval of CT may be performed by oncology nurses instead. This suggestion is supported by studies showing that nurse-led phone calls to monitor CT side effects are feasible and safe [31,32]. In fact, enhancing the role of nurses in the management of patient side effects is increasingly considered an important component of effective healthcare.

This work has several limitations that must be acknowledged. First, the study used a before–after design, as a result of which the two populations may not have been comparable, and some biases may have occurred. It should be noted, however, that randomized designs are difficult to implement for evaluating changes in the organization of care, whereas before–after designs can help demonstrate the immediate impact of a new procedure of care. Second, the monocentric nature of the study and the fact that only women with breast and gynecological cancer were included likely compromised the generalizability of our findings. Third, data about CT toxicity were not collected in the study as no difference was expected between the two groups, with the chemotherapy being the same in both cases. However, based on patient-reported data in the questionnaire, we did not observe differences in the number of consultations with the General Practitioner (GP) as well as home care services in patients included in the intervention group, suggesting that the new organizational system of CT deliverance was not associated with more toxicities. Lastly, waiting times were patient-reported, leading to a significant rate of missing data (16.3% before imputation and 6.9% after imputation). This lack of “objective” information about actual waiting times is a limit, as there may be a discrepancy between subjective and objective measures of time.

## 5. Conclusions

This work examined the implementation of a phone-based CT approval procedure for day 8 and/or 15 of the CT cycle. Our findings suggest that advanced approval of outpatient CT via phone call is a feasible alternative that does not compromise patient satisfaction with care.

## Figures and Tables

**Table 1:** Description of the sample and comparison between the two groups (control vs. intervention).

Variables	All Patients *N* = 233		Control *n* = 129		Intervention *n* = 104		*p*
	*N*	*n* (%)	*N*	*n* (%)	*N*	*n* (%)	
Age							
27–47 years	233	36 (15.5)	129	18 (13.9)	104	18 (17.3)	0.570
48–68 years		148 (63.5)		81 (62.8)		67 (64.4)	
69–85 years		49 (21.0)		30 (23.3)		19 (18.3)	
Marital status							
Living with a partner	222	132 (59.5)	123	80 (65.0)	99	52 (52.5)	0.059
Single		90 (40.5)		43 (35.0)		47 (47.5)	
Level of education							
Primary school	219	24 (11.0)	121	16 (13.2)	98	8 (8.2)	0.476
Secondary school		121 (55.2)		66 (54.6)		55 (56.1)	
University		74 (33.8)		39 (32.2)		35 (35.7)	
Professional activity							
In activity	223	99 (44.4)	125	54 (43.2)	98	45 (45.9)	0.685
No activity		124 (55.6)		71 (56.8)		53 (54.1)	
Financial situation							
Good	229	68 (29.7)	127	38 (29.9)	102	30 (29.4)	0.614
Satisfactory		105 (45.8)		61 (48.0)		44 (43.1)	
Bad		56 (24.5)		28 (22.1)		28 (27.5)	
Traumatic event impact scale (IES)							
Score < 26	190	136 (71.6)	102	70 (68.6)	88	66 (75.0)	0.331
Score ≥ 26		54 (28.4)		32 (31.4)		22 (25.0)	
Quality of life (EQ-5D)							
M (95%CI)	210	85.8 (84.6–87.0)	119	85.5 (83.6–87.3)	91	86.3 (84.8–87.8)	0.494
Cancer location							
Cervix and body of the uterus	225	23 (10.2)	123	11 (8.9)	102	12 (11.8)	0.740
Ovary		88 (39.1)		50 (40.7)		38 (37.2)	
Breast		114 (50.7)		62 (50.4)		52 (51.0)	
Chemotherapy protocol							
Mono-chemotherapy	214	123 (57.5)	112	67 (59.8)	102	56 (54.9)	0.467
Polychemotherapy		91 (42.5)		45 (40.2)		46 (45.1)	
Metastases							
No	203	58 (28.6)	105	30 (28.6)	98	28 (28.6)	1.000
Yes		145 (71.4)		75 (71.4)		70 (71.4)	
Self-reported time in the oncological outpatient unit (h)							
M (95%CI)	217	4 h 48 min (4 h 34 min–5 h 02 min)	121	5 h 10 min (4 h 44 min–5 h 22 min)	96	4 h 29 min (4 h 07 min–4 h 52 min)	0.021

CI: Confidence Interval.

**Table 2:** Factors associated with score of satisfaction with physicians—univariate analyses.

Variables	Satisfaction with Physicians	p
Age		
27–47 years	60.4 (21.0)	0.088
48–68 years	68.0 (19.7)	
69–85 years	70.4 (21.3)	
Patient group		
Control (Before intervention)	67.9 (20.7)	0.621
Intervention (After intervention)	66.5 (20.1)	
Marital status		
Living with a partner	66.3 (19.9)	0.443
Single	68.5 (20.9)	
Level of education		
Primary school	72.4 (19.0)	0.418
Secondary school	66.1 (21.1)	
University	66.6 (18.6)	
Cancer location		
Cervix and body of the uterus	70.9 (18.7)	0.127
Ovary	70.5 (20.8)	
Breast	64.8 (20.1)	
Chemotherapy protocol		
Mono-chemotherapy	65.2 (20.6)	0.070
Polychemotherapy	70.5 (19.9)	
Consultation with an oncologist before chemotherapy sessions		
Useful	68.32 (20.20)	0.104
Not useful	62.99 (20.84)	
Out-of-hospital consultation with a physician since the start of chemotherapy		
Yes	65.9 (19.8)	0.094
No	72.3 (23.3)	
Quality of life (EQ-5D)		
Correlation score	0.2	0.026
Traumatic event impact scale (IES)		
Score < 26	68.0 (19.6)	0.344
Score ≥ 26	64.7 (22.3)	
Self-reported time in the oncological outpatient unit (h)		
Correlation score	−0.03	0.668
Time spent in the oncological outpatient unit during the last chemotherapy session		
Shorter than expected	82.4 (16.9)	0.007
As expected	64.6 (20.4)	
Longer than expected	65.8 (19.1)	
Time spent in the oncological outpatient unit was adapted to the care received		
No	60.4 (19.5)	0.001
Yes	70.2 (19.9)	

**Table 3:** Factors associated with satisfaction with physicians: Multiple linear regression analysis (*N* = 170).

Satisfaction with Physicians	Coefficient	p
Age		
27–47 years	1	
48–68 years	8.2	0.062
69–85 years	10.6	0.044
Patient group		
Control (Before intervention)	1	
Intervention (After intervention)	−4.1	0.184
Self-reported time in the oncological outpatient unit (h)	−0.01	0.887
Quality of life (EQ-5D)	0.4	0.025
Time spent in the oncological outpatient unit was adapted to the care received		
No	1	
Yes	8.9	0.008

## Data Availability

Data are available upon request from the authors.
